# High‐Grade Urothelial Carcinoma Arising From a Ureteral Diverticulum: A Rare Case Report

**DOI:** 10.1002/iju5.70153

**Published:** 2026-03-02

**Authors:** Iason Papadopoulos, Stephan Ledderose, Benedikt Ebner, Elena Berg, Can Aydogdu, Julian Marcon, Robert Bischoff, Philipp Kazmierczak, Christian G. Stief, Lennert Eismann

**Affiliations:** ^1^ Department of Urology University Hospital, LMU Munich Munich Germany; ^2^ Institute of Pathology, LMU Munich Munich Germany; ^3^ Department of Radiology University Hospital, LMU Munich Munich Germany

**Keywords:** diagnostic imaging, diverticulum, hematuria, ureter, ureteral neoplasms

## Abstract

**Introduction:**

Ureteral diverticula are rare urological anomalies characterized by outpouchings of the ureteral wall. Malignancy arising from these structures is exceptionally rare, with only a few cases reported.

**Case Presentation:**

We report a 57‐year‐old male with high‐grade urothelial carcinoma arising from a ureteral diverticulum in the left distal ureter. The patient experienced painless gross hematuria, and computed tomography revealed a 3 cm undetermined mass near the left distal ureter. Multiple endoscopic diagnostic procedures were inconclusive, with the final diagnosis confirmed intraoperatively. The surgery involved resection of the mass, and intraoperative frozen section analysis revealed high‐grade urothelial carcinoma. A segmental ureteral resection was performed with reconstruction using a Boari flap and Psoas hitch. Final histopathology confirmed locally advanced, high‐grade pT3 urothelial carcinoma, and adjuvant chemotherapy was recommended.

**Conclusion:**

This case underscores the diagnostic and management challenges of upper tract urothelial carcinoma, especially in rare cases involving ureteral diverticula.

## Keynote Message

This report demonstrates the diagnostic difficulties and management challenges of urothelial carcinoma arising from a ureteral diverticulum. Early recognition of such untypical clinical presentations is critical to ensure timely and appropriate treatment. Understanding cases like this may lead to improved diagnostic strategies and better outcomes for patients with similar conditions.

## 1 Introduction

Ureteral diverticula (UD) are rare urological entities with approximately 50 cases documented in the literature. First described in 1808 by Pepper et al. during an autopsy [1], these entities can occur anywhere along the ureter but are most commonly found distally [2]. The clinical presentation of UD is variable; while many cases remain asymptomatic and are incidentally detected on imaging, other manifestations include painless gross hematuria and obstructive uropathy, often associated with ureteral stones. Additionally, UD may lead to complications such as pyelonephritis [3].

The diagnosis of UD poses significant challenges due to its rarity and variable presentation. Ultrasonography has been used in antenatal detection of UD [4], and computed tomography (CT) is commonly used in diagnosis, especially in cases involving ureteral stones, but the sensitivity of CT has yet to be reported. Retrograde ureteropyelography remains the primary modality for definitive imaging [3]. However, there is no international consensus or gold standard for diagnosing this urological anomaly.

Whereas most UD remain clinically asymptomatic and require no further treatment, a subset of UD may cause relevant complications such as perforation, hydronephrosis, or chronic infection [3]. As it is described in the lower urinary tract, diverticula harbor increased risk for meta‐ or neoplastic transformation [5].

So far, only two cases have been reported in the literature describing urothelial carcinoma (UC) arising from a UD [6, 7].

In this case report, we present a rare instance of a patient diagnosed and successfully treated at our institution for high‐grade UC arising from a UD in the left distal ureter.

## 2 Case Presentation

**FIGURE 1:**
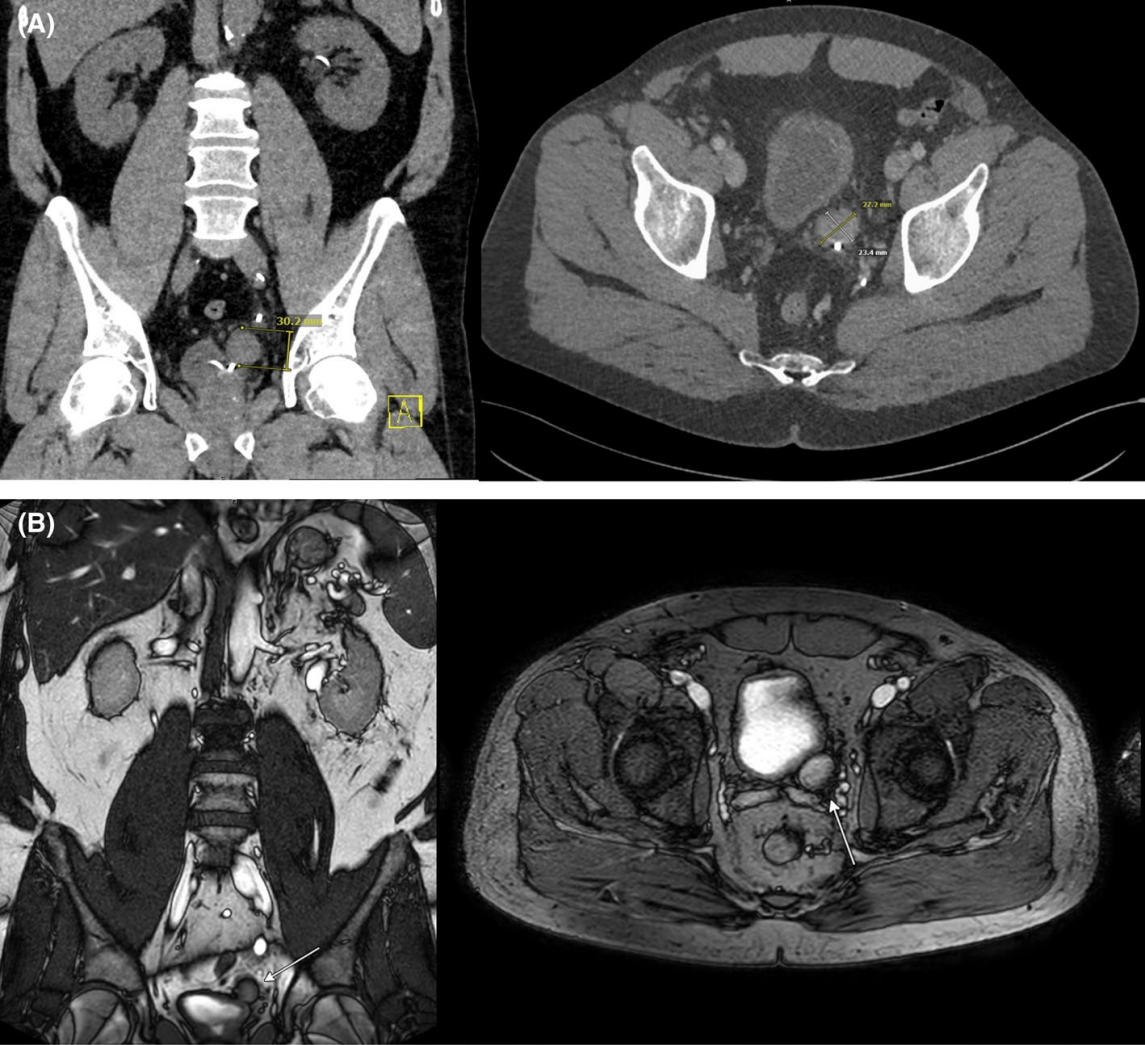
(A) Computed‐tomography and (B) magnet‐resonance imaging of the left ureteral diverticulum.

A 57‐year‐old male presented to our department with a 12‐month history of intermittent painless gross hematuria. The patient had no chronic diseases or history of urolithiasis. Initial evaluations including cystoscopy and ureteroscopy did not reveal malignancy. A subsequent contrast‐enhanced CT scan identified an indeterminate 3 cm mass adjacent to the left distal ureter (Figure 1A), which was further characterized by MRI with urography as a homogeneously enhancing mass without nodal infiltration (Figure 1B).

**FIGURE 2:**
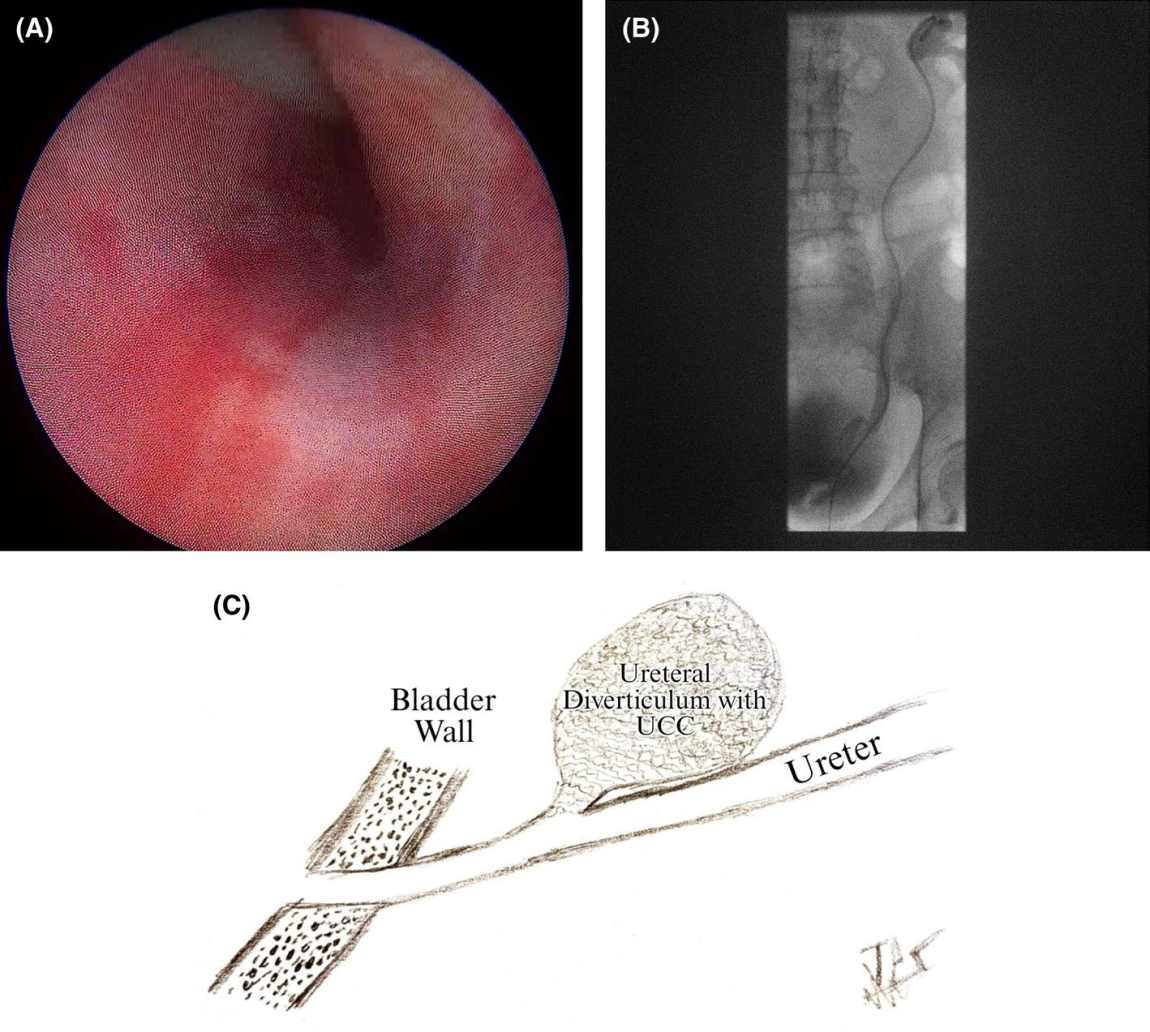
(A) Intraoperative appearance of distal ureter showing no evidence of intraluminal tumor or diverticulum. (B) Simultaneous performed retrograde urography reveals luminal communication of ureteral diverticulum and ureter. (C) Schematic illustration of ureteral diverticulum tumor.

Upon admission, the patient was asymptomatic except for microscopic hematuria detected on urinalysis. Further diagnostic efforts at our center yielded inconclusive findings. Repeat ureteroscopy revealed no intraluminal lesion, opening, or diverticulum (Figure 2A), and ureteroscopic biopsies of the distal ureter demonstrated no evidence of malignancy. Urine cytology was consistently negative for malignancy. However, retrograde ureteropyelography demonstrated subtle contrast opacification extending into a small distal ureteral diverticulum, indicating a communicating tract in this region (Figure 2B,C).

**FIGURE 3:**
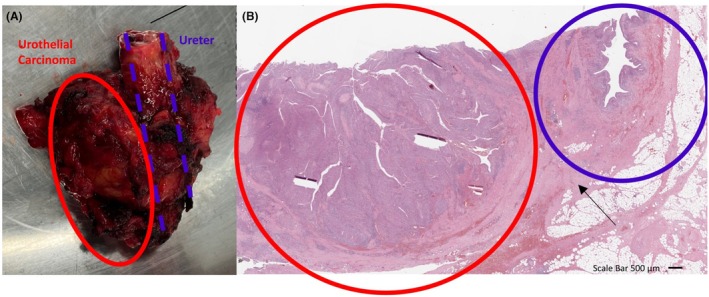
(A) The gross specimen in this panel demonstrates a segment of the ureter involved by an infiltrating urothelial carcinoma. The tumor mass (circled in red) is visibly invading and distorting the normal tissue architecture, extending towards the adjacent ureter (outlined in blue). (B) The histological section (H&E stain) highlights urothelial carcinoma within the red‐circled area, characterized by disorganized cell architecture and nuclear atypia. The tumor infiltrates through the ureteral muscularis and extends into the periureteric adventitia/fat (arrow), consistent with pT3 disease. In contrast, the blue‐circled region displays relatively normal ureteral structure, including the lumen and intact urothelium, despite being near tumor infiltration.

Due to the mass's indeterminate nature, our interdisciplinary tumor board recommended surgical exploration. The patient underwent open surgery 1 month later, where the 3 cm mass arising from the left distal ureter was isolated and excised (Figure 3A). Intraoperative frozen section analysis confirmed high‐grade UC, leading to a distal left ureterectomy including the bladder cuff, with reconstruction using a Boari flap and Psoas hitch. The estimated blood loss was 300 mL, and the procedure lasted 175 min.

Postoperative pathology confirmed high‐grade papillary urothelial carcinoma, staged as pT3. The patient's recovery was uneventful, and follow‐up CT imaging 6 weeks later showed no signs of recurrence or metastasis. Based on these findings and current guidelines for upper tract urothelial carcinoma (UTUC), adjuvant chemotherapy with Gemcitabine and Cisplatin was recommended.

## 3 Discussion

The literature on UD is characterized by ambiguous terminology, as various publications often identify different conditions under the same name. Culp aimed to standardize this in 1947 by classifying UD into three types [8]:

1. Congenital Diverticulum: Comprising all layers of the ureteral wall.
2. Acquired Diverticulum: Results from mucosal herniation due to obstructions like calculi or strictures.
3. Abortive Diverticulum: Arises from incorrect embryonic ureteral budding, often reported in literature as bifid ureter.

To complicate things further, Rank et al. [2] in 1960, argued that the differentiation between congenital diverticula (1) and abortive diverticula (3) lies on their configuration, suggesting that these terms overlap in many cases.

Histopathological examination of our case identified all layers of the ureteral wall and classified the UD in question as an overdistended bifid ureter (Figure 3B).

Another distinct but notable condition discussed in the literature is ureteral pseudodiverticulosis, often associated with UC in nearly one in two patients. Unlike ureteral diverticula, pseudodiverticulosis consists of multiple small outpouchings, typically less than 5 mm in size, often bilateral, and usually located in the upper two‐thirds of the ureter. The exact etiology remains unclear, but it is likely related to chronic inflammation or obstruction [9].

As previously noted, only two cases of UC arising from a UD have been reported in the literature. Harrison reported the first in 1983, where a congenital UD was associated with malignancy in a patient with hematuria, leading to nephro‐ureterectomy and subsequent adjuvant radiation therapy due to lymphatic invasion [6]. The second case, by Prescott in 1990, depicted an acquired UD in a patient who underwent nephro‐ureterectomy after urine cytology suggested UC, revealing two ureteral strictures and early‐stage UC in a diverticulum [7].

Furthermore, one case from 1998 was identified specifically describing pT3 UC arising from the blind end of a bifid ureter. In that case, right total nephroureterectomy was performed followed by adjuvant chemotherapy [10].

According to our knowledge, we present the first MRI and CT scan imaging of a tumor arising from a UD (Figure 1).

Moreover, neoplasms within bladder diverticula are well‐documented, with these tumors constituting about 1% of all bladder UCs. These tumors are thought to result from urinary stasis, chronic infection, and inflammation [5]. In our case of abortive/congenital UD, we hypothesize prolonged urine stasis likely led to UC development.

It is unclear whether diverticulum‐originating tumors are a distinct entity, which may require specialized treatment. Early detection remains the key challenge, highlighting the importance of experienced clinicians and specialized referral centers for optimal care.

Lastly, while radical nephroureterectomy remains the standard approach for high‐grade UTUC, isolated tumors of the distal ureter represent an important exception. In accordance with the EAU UTUC Guidelines (weak recommendation), distal ureterectomy with ureteral reimplantation is an acceptable kidney‐sparing strategy for anatomically localized distal disease [11]. Retrospective analyses suggest that oncologic outcomes are comparable to radical nephroureterectomy in this subgroup [12]. Furthermore, renal preservation may significantly influence systemic treatment eligibility, as only a minority of patients are cisplatin‐eligible following nephroureterectomy [13]. In this case, organ preservation enabled adjuvant gemcitabine–cisplatin therapy and was supported by close surveillance imaging without evidence of recurrence to date.

## 4 Conclusion

This case underscores the challenges of diagnosing and managing unusual presentations of UTUC, particularly when associated with UD. Despite multiple evaluations, including retrograde ureteropyelography and ureteroscopy, the diverticulum went undetected. Even with advanced CT and MRI scans, a definitive diagnosis remained elusive until surgical exploration. This highlights the importance of maintaining suspicion and considering surgical exploration in diagnostically ambiguous urological lesions, even when initial histology and cytology appear unremarkable.

## Consent

Written informed consent was obtained from the patient for publication of this case report and accompanying images.

## Conflicts of Interest

The authors declare no conflicts of interest.

## Data Availability

The data that support the findings of this study are available on request from the corresponding author. The data are not publicly available due to privacy or ethical restrictions.
